# Grid development for energy justice and a just transition

**DOI:** 10.1016/j.isci.2026.116762

**Published:** 2026-07-16

**Authors:** Fernando Tormos-Aponte

**Affiliations:** 1 Department of Sociology, University of Pittsburgh, 230 South Bouquet Street, Pittsburgh, PA 15260, USA; 2 Just Transition Alliance, 2615 Camino Del Rio South, Suite 400, San Diego, CA 92108, USA

**Keywords:** energy justice, just transition, grid resilience, power outages, environmental justice, Hurricane María, Puerto Rico, energy oppression, social vulnerability, energy democracy

## Abstract

The decline in the resilience of the US power grid and its territories is a matter of life and death and undermines well-being. Energy access, affordability, and reliability are central to environmental justice and key social determinants of health and mobility; yet, inequalities in grid reliability remain under-addressed. Drawing on the observations and research on the Hurricane María outages, this perspective discusses the unequal consequences of grid unreliability and argues that environmental justice and just transition principles offer fruitful ways forward. Grid-resilience efforts grounded in these approaches can reduce loss of life, economic losses, and disruptions to healthcare systems and medical supply chains. I call for targeted investments in grid development for energy-oppressed communities, closing data gaps on medical and social vulnerability, and measures to curtail energy clientelism and corruption. Grid resilience is not solely a technical and economic problem but a social one requiring an environmental justice approach.

## Graphical abstract

**Figure:**
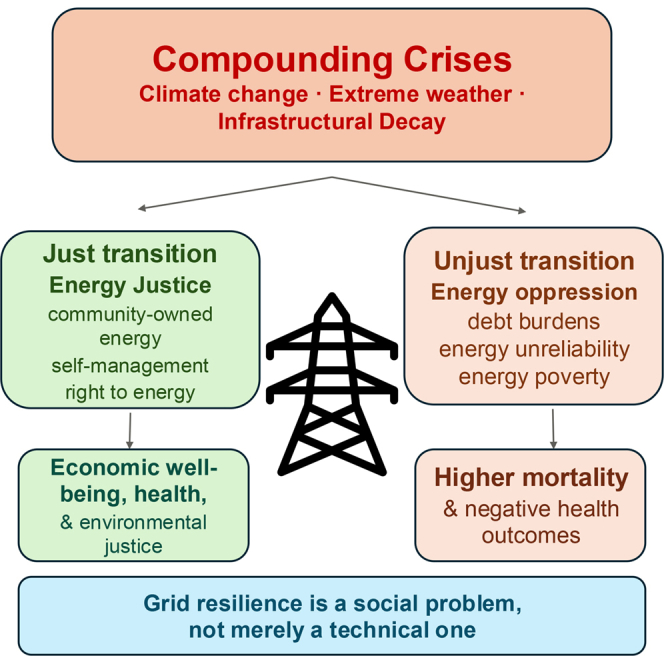

## Teaser

Energy policy; Energy management; Sociology

## Introduction

Climate change makes extreme weather, the most common cause of power outages,^1^ more frequent and intense,^2^ prolonging outages and increasing their frequency.^3^ Outages have increased significantly over the past 10 years, exposing US electricity consumers to nearly 7.5 h of power outages per year.^4^ The decline in the resilience of the US power grid and its territories is a matter of life and death and undermines well-being. Greater attention to inequalities in grid reliability and energy resilience is needed, as energy access, affordability, and reliability are central to economic and environmental justice and are key social determinants of health and social mobility.

Grid reliability refers to the provision of an adequate, secure, and stable flow of electricity to meet consumer demand.^5^ The National Renewable Energy Laboratory^6^ identifies three core components of reliability: (1) resource adequacy, which refers to the ability of the system to supply aggregate electricity demand at all times; (2) operational reliability, which refers to the ability to withstand sudden disturbances while avoiding cascading failures; and (3) resilience. While reliability focuses on maintaining consistent service, resilience captures the grid’s capacity to cope with disruptions. The Federal Energy Regulatory Commission^5^ describes resilience as encompassing the attributes and services that enable the grid to withstand, adapt to, and recover from both natural and man-made disruptive events. The National Infrastructure Advisory Council^7^ posits that effective resilience depends on a system’s ability to anticipate, absorb, adapt to, and rapidly recover from potentially disruptive events.

Energy is the lifeblood of the global political economy and its supply chains. Efforts to increase grid reliability in the context of increasing intensity and frequency of climate change-related extreme weather events have global implications, as shocks to grids in one region can have global ripple effects.^8^ For instance, the Hurricane María outage, the longest outage in US history, disrupted local health systems and global medical supply chains, which relied on Puerto Rico’s manufacturing industry for 8% of medicines consumed in the US.^9^

Disruptions in medical care, often caused by outages, are among the primary drivers of post-disaster mortality. Such was the case following Hurricane María in Puerto Rico. Outages are associated with mortalities,^10^^,^^11^^,^^12^ mental illness,^13^ and economic losses among individuals, industries, and commercial entities.^8^^,^^14^^,^^15^^,^^16^ Even seemingly brief disruptions of the electricity supply can cause multi-billion-dollar economic losses.^17^ Bhattacharyya, Yoon, and Hastak find that even a 1% inoperability in the US utility sector would cause an estimated $11.6 billion losses in GDP (in 2019 dollars).

This perspective discusses the inequality in grid reliability and the unequal nature of consequences that result from a lack of reliability. Further, I argue that environmental justice and just transition principles point a fruitful way forward for grid development. Grid development refers to the planning, expansion, modernization, and reconfiguration of electricity infrastructure, including generation, transmission, distribution, and storage. I draw on my observations and research on Hurricane María outages to reflect on the challenges posed by grid unreliability and to consider how grid resilience efforts grounded in environmental justice and just transition approaches can reduce loss of life, economic losses, and disruptions to healthcare systems and medical supply chains. I conclude with suggestions for enacting energy policy change that advance energy justice and a just transition. Namely, I call for targeted investments in grid development and resilience for energy-oppressed communities, addressing data gaps on medical and social vulnerability, improving data use, and adopting measures to curtail energy clientelism and corruption (see the Committee on the Future of Electric Power^18^ for greater insight into grid development in the US). Further, I argue that the pursuit of grid resilience is not solely a technical and economic problem, as current energy management practices and policies suggest, but a social one that must address energy inequalities and marginalization through an environmental justice approach.

## Energy justice and grid resilience

Grid unreliability has far-reaching impacts. These impacts disproportionately affect the already marginalized communities,^18^ including energy-oppressed communities who are often the last to receive electricity and spend the longest time without power. Marginalization is a condition in which social groups face disadvantages across various aspects of life, including social, political, and economic relations, leading to exclusion from societal institutions and challenges in meeting their needs.^19^^,^^20^^,^^21^ Marginalization can manifest as the obstruction to resources controlled by dominant groups,^19^ such as energy. I define energy oppression as the systematic obstruction of access to reliable energy for social groups. This definition extends the notion of marginalization^21^ to the energy domain, where dominant groups control a resource that disadvantaged communities are excluded from accessing on equal terms. In the context of grid reliability, energy oppression manifests across several dimensions: who has access to electricity and the duration, frequency, and severity of the outages they experience when service is disrupted.

The extent of energy marginalization is not well understood due to data barriers and other factors that impede a deeper understanding of this problem, including ongoing attacks on scientific integrity and scientific institutions, the proliferation of misinformation, and the erosion of democratic norms. For example, the second Trump administration undermined the efforts to study and address energy marginalization, including measures such as terminating the Climate and Economic Justice Screening Tool (CJEST), sidelining the environmental health role of the Centers for Disease Control and Prevention (CDC), defunding environmental justice research, and rescinding clean energy grid resilience grants.

Various features of energy inequality and justice merit attention to deepen the understanding of the barriers and pathways to grid resilience. The pursuit of grid resilience through environmental justice and just transition approaches must be attentive to the systemic dynamics of inequality and justice, social group disparities, ecological destruction and its impact on human and non-human species, political injustices of exclusion from decision-making processes, economic inequalities, and the failure to recognize marginalized groups as rightsholders.^22^^,^^23^^,^^24^ Tormos-Aponte, Wright, and Brown^25^ provide a discussion of these social structures and systems. Just transition has been defined as a series of ideas, principles, and processes that aim to involve groups that have been adversely affected by efforts to transition away from fossil-based economies in decision-making and policymaking as well as to provide compensation for those affected groups.^23^ The pursuit of energy justice has intellectual roots that have been traced back to ancient political theory and contemporary theories of justice.^24^ Rich debates on the political theories of justice, often reflecting on and interacting with social movements pursuing justice, suggest that justice must include the recognition of those impacted by decision-making processes and sociopolitical systems, the representation of those groups in those systems and decision-making processes, and the redistribution of resources concentrated in the hands of dominant groups to meet the needs of marginalized groups.^26^^,^^27^^,^^28^^,^^29^^,^^30^

Perhaps the most prominent and politically influential articulations of environmental justice have emerged from social movements. These movement-generated notions of justice provide guidance for grid development. Following a series of activist campaigns to resist toxic waste dumping, court cases, and studies documenting the disproportionate exposure of ethnoracially and economically marginalized communities to pollution,^31^^,^^32^^,^^33^^,^^34^^,^^35^ civil rights, labor, Indigenous, and environmental organizers and organizations gathered in Washington, DC, for the First National People of Color Environmental Leadership Summit in 1991. This meeting concluded with the publication of the Principles of Environmental Justice.^36^ Subsequently, alliances between oil and gas industry labor unions and fenceline communities (communities in the vicinity of industrial facilities) laid the groundwork for the development of the principles of a just transition.^22^^,^^23^^,^^37^^,^^38^ These efforts built on histories of justice, labor, and human rights activism and provided opportunities to continue advancing movement-generated theories, ideas, and perspectives all too often neglected in academic writing and more often finding an intellectual home in the Black radical, feminist, and decolonial traditions.

Collectively, environmental justice and just transition principles have important implications for achieving grid resilience and equity. These principles call for recognizing the sacredness of Mother Earth; the end of all destructive corporate activities; the right to be free from pollution and ecological destruction; the enforcement of the principle of free, prior, and informed consent as part of the recognition of the collective rights of Indigenous Peoples; the right to self-determination; the enactment of policy free from discrimination; the right to participate in decision-making processes and policymaking; affirming the rights of workers; and calling for the compensation of those impacted by histories of environmental destruction and the consequences of transitioning away from fossil fuel-based systems (among various other demands). In this view, achieving these aims would require a systemic transformation of the global political economy while also assuming collective responsibilities for reducing energy consumption. With these principles and concerns over justice and equity in mind, grid resilience efforts must adopt a lens to understand the scope of the problem, pursue courses of action to address it, and track their progress.

## The unequal consequences of grid unreliability

Grid unreliability stems from multiple interconnected factors. A primary driver is the neglect of aging infrastructure by governments and utility companies, which can take both intentional and unintentional forms. Neglect includes the corrupt, clientelistic distribution of energy and grid-related resources^39^ as well as policy inaction or insufficient investment in maintenance and modernization. These vulnerabilities are compounded by the growing stress that climate change and extreme weather place on the built environment. As electricity demand rises and outpaces supply, communities face increasing energy poverty (see Bednar and Reames^40^) and exposure to rolling blackouts through load shedding, which is the practice of imposing discretionary, planned outages to balance supply and demand and prevent larger, grid-wide failures.

Unequal grid outcomes do not arise from a single source. They emerge from a combination of unconscious biases in how energy operations are designed and administered, intentional political choices about where to invest, and systemic inequalities embedded in institutions themselves. While some biases in energy operations may be unconscious and unintentional, there are also intentional instances of government neglect whereby energy resources are distributed for electoral gain.^39^ Marginalized communities tend to elicit less government responsiveness and grid investment, often due to their low tax bases^41^ and perceptions among elected officials that investing in these communities carries low electoral rewards.^42^^,^^43^ Investments in disaster preparedness, including grid resilience, are less attractive to politicians than post-disaster aid distribution,^44^^,^^45^ which allows them to stage more visible performances of government responsiveness and capable governance. The visibility of government investments enables electoral rewards for incumbents.^46^ Despite its vital role in sustaining life and societies, the provision of electricity may be a resource that voters take for granted and do not reward the incumbents for ensuring grid reliability. Thus, grid development policies face the challenge of mobilizing support among elected officials who may lack electoral and financial incentives to support those most impacted by grid unreliability.

Systemic inequalities do not only stem from intentional acts. Scholars of racism have argued that racism is a systemic problem resulting from institutionalized practices and institutional designs as well as networks of formal and informal norms that shape social relations and enable the domination of some groups over others.^47^ Thus, these systemic inequalities structure human interactions such that intentional efforts to enact discriminatory courses of action are not needed to engender discriminatory outcomes and inequalities across social groups.^25^ The relegation of energy decision-making to an arena of technical concerns creates conditions for the exclusion of public participation and the introduction of biases into energy resource allocations in ways that are rarely subject to public scrutiny.

Electric utility officials tend to dismiss non-technical concerns in grid operations.^48^ In the interviews I conducted with emergency operations and utility officials in Texas, Florida, and Puerto Rico following the devastating 2017 hurricane season, respondents often dismissed the possibility of paying attention to social group disparities (such as inequalities across racial or economic class groups), proudly claiming that their job was a technical one that followed established industry protocols. However, the discretionary elements of grid operations, particularly during emergencies and during supply shortages, introduce biases into policy implementation. Further, the industry “best practice” of prioritizing densely populated regions in power restoration processes—the density-based approach^48^—makes rural and remote regions particularly vulnerable to outages by design.^49^ Following Hurricane María, remote and predominantly Afrodescendant regions also experienced delays in the deployment of restoration crews and, thereby, in energy restoration.^48^^,^^50^^,^^51^ During prolonged outages like those caused by Hurricane María, generators become a life-sustaining resource. In a remote community in Lajas, I learned that a former community leader had to keep a generator operating continuously for months to power her mother’s electricity-dependent medical equipment, yet generators are often financially out of reach for economically marginalized communities. Further, risk information asymmetries often expose groups to carbon monoxide poisoning,^52^ including those who feel compelled to bring their generators indoors due to fear of theft.

The consequences of grid unreliability extend beyond economic losses to encompass serious public health outcomes, including disruptions in health systems, global medical supply chains, healthcare visits, mental health impacts, and elevated mortality rates.^11^ These consequences are not evenly distributed across social groups. Studies using metrics such as the Gini coefficient and median outage duration have revealed significant social and spatial disparities in both managed and hazard-induced power outages, with marginalized communities bearing disproportionate burdens.^48^^,^^53^ The consequences of grid unreliability fall disproportionately on medically vulnerable populations. Approximately 14.5 million US households rely on electricity-dependent medical devices and nearly a third of these households experienced power outages in a 12 month period.^54^ The health consequences can be severe: people with home medical devices accounted for roughly one in five hospital admissions in the 24 h following the 2003 New York blackout and planned load shedding events in South Africa have been linked to a 10% increase in hospital admissions from both medical device failures and secondary effects such as carbon monoxide poisoning and food spoilage.^55^^,^^56^

Current electrical utility power restoration policies follow a two-tier priority system: essential services are restored first, followed by areas where each crew deployment can reconnect the largest number of consumers.^48^ Thus, neglecting non-technical concerns, such as the disproportionate exposure of marginalized communities to outages, may exacerbate existing disadvantages by design. This characterization does not imply intentionality, that is, that the individuals involved in designing these standards intended to discriminate against marginalized groups; rather, the unequal outcomes of seemingly neutral policies are a well-known feature of racism.^47^ The US Department of Energy outlines these priorities in its step-by-step power restoration process. These biases, combined with uninterrupted and ongoing legacies of inequality and marginalization, disproportionately expose marginalized communities to grid unreliability. In this context, it is no surprise that communities that rank among the highest in their social vulnerability and have the highest concentration of people relying on electricity-dependent medical equipment are among the most affected by outages.^57^ The consequences of grid unreliability underscore the need for large-scale investments to meet energy needs. Yet, despite their vitality for sustaining life and the systems on which livelihoods depend, the development and maintenance of electrical power grids are failing to keep pace with existing and projected needs.^58^

Climate change and extreme weather motivate the need for grid resilience investments as part of broader climate change adaptation measures. A failure to mobilize resources for these investments disproportionately exposes marginalized groups to loss and damage, thereby exacerbating historical inequalities and deepening environmental injustices. These historical inequalities include US government-sponsored planning efforts that prioritized grid development in the service of industrial and military interests while inconsistently enabling energy access, affordability, and reliability in disadvantaged communities exposed to infrastructural vulnerabilities.^59^ Electrification efforts have yet to reach the goal of providing electricity to all. As of 2020, more than 800 million people, mostly in the Global South and in rural areas, remained without electricity.^60^ These policy failures continue despite the potential for grid reliability to reduce loss of life. Further, grid reliability has the potential to narrow wealth gaps and advance economic well-being.^61^

## The role of grid resilience in environmental justice and just transition

Energy grid investments are needed to achieve global, systemic, and just transitions away from fossil-fuel dependence through the distribution of renewable energy. In making these investments, grid resilience helps reduce the vulnerability of marginalized communities to climate change, extreme weather-related loss and damage, and the harmful consequences of outages. Grid development is needed to increase electrification and enable greater access to renewable energy, free from ecological destruction, human rights violations, and corruption. Grid development can also be a pathway to reduce reliance on centralized, top-down governance of energy resource management, enabling greater community involvement in energy policymaking.

Energy grid development is at a crossroads. One path further entrenches energy infrastructure as a means for wealth accumulation and endless economic growth. An alternate path pursues grid development as a means of advancing the right to clean energy and a just transition, which can be realized under a series of conditions, including the mobilization of social forces for policy change. These pathways can co-exist in contradictory and complex ways. An alternative, justice-centered path is grounded in a broader tradition of social change efforts aimed at pursuing environmental justice. Social change of this magnitude, particularly in the context of inconsistent political and economic incentives for elected officials, may require mass political advocacy. Social movements are strong forces for social change, particularly when paired with openings in the political climate, supportive views within the electorate and the mass public, and the adoption of diverse tactics, among other conditions that scholars of social change identify as enablers of large-scale societal transformations.^62^ Scientists also have an important role in advancing social change.^63^ Collectively, social change efforts have the potential to hold polluters accountable for their historical harms, move them to assume responsibility for achieving justice, and enable compensation for those negatively impacted by climate change.

The process of systematically transforming grid infrastructures to achieve grid resilience and environmental justice is as important as the outcome itself. The inclusion of those impacted by decision-making processes is often referred to as procedural justice. Grid resilience will be at odds with just transition and environmental justice principles if it does not emerge from participatory, inclusive, and democratic decision-making processes, compensate those negatively impacted by these transformations, and respect the rights of marginalized groups. These rights are distinct across groups, and they include the right to energy. Further, the achievement of grid resilience must avoid harms against groups resulting from the reliance on the extraction of critical minerals for clean energy without the free, prior, and informed consent of Indigenous Peoples and other impacted communities. The role of inclusion and social movements in the collective decision-making process for resource allocation is associated with enhanced resource management and innovation.^64^ Social movements, in their efforts to achieve social change, must also project the values they claim to stand for to increase their legitimacy in the public eye. This means adhering to principles of inclusive and democratic organizing, such as the Jemez Principles of Democratic Organizing, and adopting intersectional solidarity approaches to organizing.^65^^,^^66^ In doing so, social movements enhance democratic representation in policymaking and are better positioned to move state actors to action, which must take on a leadership role in advancing national just transition pathways.^20^^,^^67^ Further, Global North states are under greater pressure to lead in the provision of funding to enable just transitions, given their historical contributions to climate change.

Empirical cases demonstrate that justice-centered grid development is achievable and that social movements have been central to advancing it. In Adjuntas, Puerto Rico, community organizations and local businesses responded to the prolonged failure of centralized grid recovery after Hurricane María by building a cooperatively managed solar microgrid that now sustains critical services during outages, while at the federal level, the US Department of Energy’s Grid Resilience and Innovation Partnerships program briefly required Justice40 commitments in all funded grid investments before being rolled back under subsequent administrative reversals. These outcomes reflect a broader pattern documented in the energy democracy and energy justice literatures, in which grassroots and environmental justice movements have pressured utility companies, regulators, and policymakers to redistribute decision-making authority and material benefits within energy infrastructure.^68^^,^^69^^,^^70^

## Considerations for an environmental-justice-centered pursuit of grid resilience

Efforts to address grid development needs must account for historical and ongoing environmental inequalities in grid resilience investments. These efforts would benefit from adopting holistic approaches that account for the demographic, spatial, inter-species, and temporal dimensions of energy inequalities^24^ as well as principled efforts guided by energy and environmental justice principles.^36^ Further, grid development policy should recognize energy as a human right and prioritize the provision of energy to communities disproportionately exposed to risks associated with power outages in the development of energy infrastructures. To this end, there is a need to increase investments in monitoring energy-related social and medical vulnerabilities at hyperlocal and regional scales. There is a widely recognized need to improve data collection and the use of medical and social vulnerability data.^11^^,^^71^ Data access, however, is increasingly contentious and is undermined by the proliferation of misinformation, attacks on scientific integrity, and the deterioration of democratic norms. Thus, the collection and use of scientific evidence and of social and medical vulnerability will be subject to contentious political processes and may reflect the broader political climate. Given these data gaps and barriers to using existing medical and social vulnerability data, advocacy and community-based participatory research efforts may be necessary to advance energy justice and grid reliability. In the context of Puerto Rico post-Hurricane María, civil society and legal mobilization efforts enabled investigative reporting and research that revealed that the death toll was much higher than what local and federal governments had admitted.^12^^,^^39^^,^^48^^,^^72^^,^^73^^,^^74^ Data transparency and disaster mismanagement motivated broader organizing efforts that ultimately reconfigured and infused the landscape of civil society organizations involved in advancing environmental and energy justice in Puerto Rico. The fight for data access and justice will be an important determinant of the extent to which grid developments can meet environmental justice and just transition aims.

Data barriers are among the reasons that prevent a more comprehensive understanding of energy inequalities. Despite widespread medical data collection in developed countries, medical data are more likely to be exploited for financial gain than used to sustain life in disaster settings.^75^ In this context, community organizations can engage in community data collection and stewardship efforts that enable data use for life-sustaining energy sovereignty investments and resource management, as is the case in the community of Corcovada, Añasco, where grid unreliability and government failures to provide consistent water motivated the community to develop and manage a solar-powered community aqueduct.^76^ Local governments can also engage in efforts to collect data that enables a clear understanding of where they have concentrations of medically vulnerable residents. Furthermore, existing national-level databases of medically vulnerable people, such as EmPower, must be used to inform energy grid planning decision-making. Access to granular-level EmPower data (below the county scale) is limited to local governments that can request it. However, few pursue this access and receive the support needed to use these data effectively.

Greater use of data on medical vulnerability to outages requires investment in the development of local capacities to use these data, given that few local governments and health departments know about or know how to use them.^77^ The use of these data can inform efforts to adopt an affirmative approach to grid development resource allocations that reduces the burden of powerlessness on the marginalized, questions the dominance of density-based approaches to energy restoration, and pursues more equitable and optimal approaches to load-shedding in emergency scenarios in ways that reduce loss of life and adversely impact the livelihoods of marginalized groups.

## Conclusion

Power outages and the broader impacts of grid unreliability are concentrated among marginalized communities. Energy-oppressed communities frequently experience prolonged and frequent outages due to infrastructural vulnerabilities and neglect by government and utility companies. Grid unreliability enables the use of bureaucratic discretion in energy supply decision-making, which can introduce bias and foster systemic forms of inequality and corruption. For energy to be considered clean, it must also be clean of corruption, discriminatory practices, and human rights violations in the sourcing of minerals used to generate renewable energy.

Rural and remote communities are exposed to the highest risks associated with outages. They elicit less attention from utility companies and governments, which often prioritize energy restoration in densely populated regions, guided by various technical logics. While these technical concerns are not to be dismissed, what is often overlooked is how the exclusive focus on the technical logics of power restoration further marginalized already-burdened communities. Affirmative restoration and grid resilience approaches that prioritize energy resource allocations for energy-oppressed communities hold the potential to reduce loss of life and negative impacts on well-being.

Grid resilience is necessary to achieve electrification and address the causes and consequences of climate change. Thus, achieving grid resilience is a necessary condition for the right to energy and for effective, just transitions away from fossil-fuel dependence. In the view of environmental justice coalitions historically involved in the advancement of just transition in the US, achieving a just transition that transforms the political economy of energy requires a system change through a global shift away from harmful extractivist systems toward regenerative economies that prioritize health, food sovereignty, and energy democracy (among various other considerations).^23^ This involves decommodifying energy resources so that access to energy is not subject to market fluctuations.^78^

The development of energy transmission and generation capabilities, guided exclusively by profit-seeking motives at the expense of livelihoods and well-being, risks locking communities and societies into a path-dependent logic of development that constrains future possibilities for achieving a just transition and upholding the right to energy. Capitalist, profit-driven logics of grid development can be observed in speculative investments in energy generation capacity for the artificial intelligence (AI) industry, despite community opposition and through public subsidies that reduce energy burdens on corporations while increasing energy poverty. This approach to grid development stands in contrast to resilience- and equity-driven approaches. In the past, the petrochemical industry was promoted as an engine of economic development for energy-oppressed communities, and yet, that petrochemical dream did not materialize.^79^ When AI ceases to be a widely touted engine of economic growth and development, the energy infrastructure built to support its data centers will remain in place, no longer attracting the investments it once did. As that infrastructure reaches the end of its useful life, it will fail to sustain life within communities that remain. Social change efforts are needed to engage in policymaking that views grid development as a mechanism to sustain life, ignite social mobility, support locally and democratically governed food and water systems, and enable thriving economies.

Addressing energy oppression demands grounding grid development in environmental justice principles and treating energy as a human right. It requires expanding the collection, transparency, and use of medical and social vulnerability data, and it calls for policies that allocate grid resilience resources equitably and make load-shedding decisions with principles of justice in mind, thereby reducing the losses and damages that marginalized communities are forced to bear.

## Acknowledgments

The author thanks Christopher Norris Silva and Andrew Hill for their research assistance. The author also thanks the 10.13039/100001345Social Science Research Council DRIP working group under Alexa Dietrich’s leadership and the 10.13039/100006734Princeton University/CENTRO Mellon Foundation Post-Disaster Futures study group for fruitful engagement. Further, the author thanks the 10.13039/100007921University of Pittsburgh 10.13039/100007295Mascaro Center for Sustainable Innovation, Pitt Momentum Funds, the American Political Science Association Centennial Center, the 10.13039/100006636University of Maryland, Baltimore County Center for Social Science Research, 10.13039/100000192NOAA 10.13039/100017299CESSRST, the Southern Methodist University Tower Center, the 10.13039/100008618University of Colorado Natural Hazards Center, and the NSF National Center for Atmospheric Research’s Early-Career Faculty Innovator Program (US National Science Foundation under cooperative agreement no. 1852977) for their generous support.

## Author contributions

F.T.-A. conceived the perspective and wrote the manuscript.

## Declaration of interests

The author declares no competing interests.
